# Cost‐Effectiveness of Pharmacologic Therapies for Metabolic Dysfunction–Associated Steatohepatitis With Significant Fibrosis in the United States

**DOI:** 10.1111/dom.71020

**Published:** 2026-06-25

**Authors:** Ali A. Abdeen, Turgay Ayer, Aanan Biswas, Chase J. Wehrle, Sobia N. Laique, Ali Aminian

**Affiliations:** ^1^ Department of Industrial and Systems Engineering Georgia Institute of Technology Atlanta Georgia USA; ^2^ Information Systems and Operations Management Department Kuwait University Al‐Shadadiya Kuwait; ^3^ Division of Liver Transplantation Cleveland Clinic Cleveland Ohio USA; ^4^ Division of Gastroenterology and Hepatology Cleveland Clinic Cleveland Ohio USA; ^5^ Bariatric and Metabolic Institute, Department of General Surgery Cleveland Clinic Cleveland Ohio USA

**Keywords:** cost‐effectiveness, fatty liver disease, GLP‐1, GLP‐1 analogue, liver

## Abstract

**Aims:**

The approval of new pharmacotherapies for metabolic dysfunction‐associated steatohepatitis (MASH) presents a critical need for value assessment. We evaluated the cost‐effectiveness of resmetirom, semaglutide and tirzepatide for U.S. adults with MASH and F2–F3 fibrosis.

**Materials and Methods:**

We developed a Markov cohort model with a lifetime horizon from the perspective of a U.S. healthcare payer. The model simulated biopsy‐confirmed patients with MASH progressing through liver‐specific health states. Efficacy inputs for fibrosis regression and MASH resolution were obtained from a Bayesian network meta‐analysis. Each pharmacotherapy was compared individually against standard of care; a formal sequential incremental analysis across active therapies was not performed. Cost‐effectiveness was assessed against the standard of care using a $100 000/QALY willingness‐to‐pay threshold.

**Results:**

In the base‐case analysis, tirzepatide had the largest QALY gain and the lowest incremental cost relative to standard of care, yielding an incremental cost‐effectiveness ratio of ($42 705/QALY). Semaglutide was also cost‐effective (ICER: $80 076/QALY). Resmetirom (ICER: $273 445/QALY) exceeded the WTP threshold. Drug price was the most influential parameter in sensitivity analyses; probabilistic sensitivity analysis showed a 99.5% probability of cost‐effectiveness for tirzepatide and 89.8% for semaglutide at $100 000/QALY.

**Conclusions:**

At current U.S. prices, tirzepatide and semaglutide are cost‐effective for MASH with F2–F3 fibrosis, while resmetirom is not. The tirzepatide finding should be interpreted with caution, given that it is not yet FDA‐approved for MASH and its effect estimate is based on a network meta‐analysis of a phase 2 trial. Payer coverage and equitable access to MASH therapies require value‐based pricing strategies.

## 1 Introduction

Metabolic dysfunction–associated steatotic liver disease (MASLD) affects approximately 38% of adults worldwide and 7%–14% of children and adolescents, representing a major global public challenge. Global prevalence is projected to increase to 55% by 2040 [1]. Approximately 20% of individuals with MASLD develop metabolic dysfunction–associated steatohepatitis (MASH), a progressive inflammatory phenotype that can lead to hepatic fibrosis, cirrhosis, hepatocellular carcinoma (HCC) and liver‐related death [2, 3]. MASH is now the second leading indication for liver transplantation in the United States. The associated economic burden is significant, including high direct medical costs and indirect expenses from lost productivity and decreased quality of life [4]. Without effective intervention, MASH progresses to advanced fibrosis and cirrhosis, which are strongly associated with major adverse liver outcomes (MALOs) and mortality. Halting or slowing fibrosis progression at the F2 or F3 stage is essential to improve outcomes and reduce disease‐related costs.

Although lifestyle modification, often supplemented by off‐label pharmacotherapy, is typically used as a first‐line therapy for MASH, it seldom leads to histologic fibrosis regression and is insufficient for many patients. Metabolic and bariatric surgery (MBS) offers the greatest demonstrated reduction in MALO, liver transplantation, and mortality among patients with obesity and MASH [5, 6]. Nevertheless, population‐level adoption has been constrained by procedural invasiveness, referral barriers, and limited payer coverage. Recently, pharmacologic treatments have shown promising results for histological improvement of MASH. In 2024, resmetirom was approved as the first pharmacotherapy for patients with MASH specifically at stages F2 and F3, followed in 2025 by FDA approval of semaglutide for the same indication. In addition, tirzepatide has shown a promising clinical outcome in its Phase 2 trials [7].

Despite promising clinical benefits, cost remains a significant barrier to adoption. Resmetirom's list price of approximately $135 per day could pose challenges for payer coverage and may result in substantial out‐of‐pocket costs for patients, although this topic is yet understudied. While resmetirom, semaglutide and tirzepatide are all promising pharmacologic therapies for delaying adverse liver outcomes for patients with MASH stages F2–F3, this study aimed to assess the long‐term cost‐effectiveness of each agent compared with standard of care. The objective was to inform payer decision‐making and guide equitable access to therapies that aid in delaying hepatic disease progression in high‐risk populations.

## 2 Methods

### 2.1 Model Overview

**FIGURE 1:**
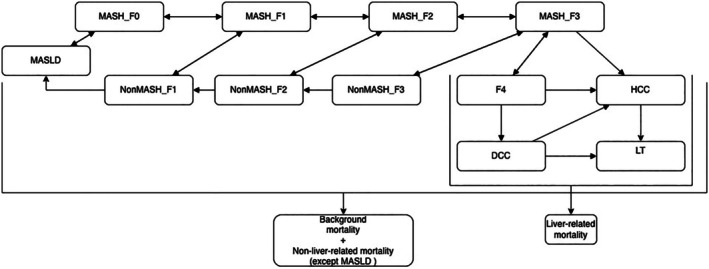
State‐transition model of MASLD and MASH progression to advanced liver outcomes. This figure presents the state‐transition framework used to model the progression of MASLD and MASH across fibrosis stages and into advanced liver outcomes including DCC, HCC, and LT. The diagram reflects annual transitions between disease states, including both progression and regression pathways, and incorporates liver‐related and background mortality risks. DCC: Decompensated cirrhosis, HCC: Hepatocellular carcinoma, LT: Liver transplantation, MASLD: Metabolic dysfunction–associated steatotic liver disease, MASH: Metabolic dysfunction–associated steatohepatitis.

We developed a Markov cohort‐based model to compare each of the three pharmacotherapies (resmetirom, semaglutide and tirzepatide) individually with standard of care (SoC) for a hypothetical cohort of U.S. adults with biopsy‐confirmed MASH with fibrosis stage F2–F3. The model incorporated 14 mutually exclusive health states to capture liver disease progression and regression: MASLD, non‐MASH (F1–F3), MASH with fibrosis states (F0–F3), F4 (compensated cirrhosis), decompensated cirrhosis (DCC), hepatocellular carcinoma (HCC), liver transplant (LT), post‐LT and absorbing death state (background mortality, liver‐related, non‐liver‐related mortality (NLRM) related to mortality due to cardiovascular events or extrahepatic malignant neoplasm) as shown in Figure 1. The model started with MASH F2 and F3 states, reflecting the current approved indication, and was simulated over a lifetime horizon using 1‐year cycles. The analysis was conducted from the U.S. healthcare payer perspective, with both costs (in 2024 US$) and quality‐adjusted life‐years (QALYs) discounted at an annual rate of 3%. The model was implemented in Python and adheres to the Consolidated Health Economic Evaluation Reporting Standards (CHEERS) guidelines [8]. Table 1 shows Key model inputs for base‐case and sensitivity analyses.

**TABLE 1:** Key model inputs for base‐case and sensitivity analyses. This table summarises the model input parameters along with the sensitivity analysis range, distribution and source. AE, adverse event; DCC, decompensated cirrhosis; F0–F4, fibrosis stages 0–4; HCC, hepatocellular carcinoma; LT, liver transplantation; MASLD, metabolic dysfunction–associated steatotic liver disease; MASH, metabolic dysfunction–associated steatohepatitis; QALY, quality‐adjusted life‐year; RR, relative risk; SoC, standard of care.

Parameter	Base‐case	Range	Distribution	Source
Initial distribution				
F2	0.5	(0.4, 0.6)	Beta (*α,β*)	Assumption
F3	0.5	(0.4, 0.6)	Beta (*α,β*)	Assumption
Annual transition probabilities (SoC)				
MASLD to MASH F0	0.1438	(0.1095, 0.1848)	Beta (*α,β*)	9
MASH F0 to MASLD	0.1000	(0.0600, 0.1400)	Beta (*α,β*)	9
MASH F0 to MASH F1	0.1336	(0.1000, 0.1700)	Beta (*α,β*)	9
MASH F1 to MASH F0	0.0810	(0.0593, 0.1095)	Beta (*α,β*)	9
MASH F1 to MASH F2	0.1352	(0.1000, 0.1700)	Beta (*α,β*)	9
MASH F2 to MASH F1	0.0810	(0.0593, 0.1095)	Beta (*α,β*)	9
MASH F2 to MASH F3	0.1413	(0.1000, 0.1800)	Beta (*α,β*)	9
MASH F3 to MASH F2	0.0810	(0.0593, 0.1095)	Beta (*α,β*)	9
MASH F3 to F4	0.095	(0.076, 0.114)	Beta (*α,β*)	Calibrated
MASH F3 to HCC	0.005	(0.004, 0.006)	Beta (*α,β*)	Calibrated
F4 to MASH F3	0.1428	(0.0860, 0.1861)	Beta (*α,β*)	9
F4 to DCC	0.0411	(0.0410, 0.0795)	Beta (*α,β*)	9
F4 to HCC	0.0141	(0.0062, 0.0378)	Beta (*α,β*)	9
DCC to HCC	0.0711	(0.0529, 0.0864)	Beta (*α,β*)	9
DCC to LT	0.0230	(0.0173, 0.0288)	Beta (*α,β*)	9
HCC to LT	0.0400	(0.0300, 0.0500)	Beta (*α,β*)	9
MASH resolution	0.08	(0.064, 0.096)	Beta (*α,β*)	9
MASH relapse	0.05	(0.04, 0.06)	Beta (*α,β*)	9
Non‐MASH one stage improvement (F3 to F2, F2 to F1, and F1 to F0)	0.0810	(0.0593, 0.1095)	Beta (*α,β*)	9
Mortality				
Annual Liver‐related rates				
DCC	0.0858	(0.06864, 0.10296)	Beta (*α,β*)	9
HCC	0.4860	(0.4794, 0.5532)	Beta (*α,β*)	9
One‐year after LT (from DCC)	0.1046	(0.0951, 0.1141)	Beta (*α,β*)	9
One‐year after LT (from HCC)	0.1232	(0.1082, 0.1401)	Beta (*α,β*)	9
*Annual* Non‐liver‐related rates				
DCC	0.0526	(0.0224, 0.1297)	Beta (*α,β*)	9
HCC	0.0800	(0.0760, 0.1196)	Beta (*α,β*)	9
LT (from DCC)	0.0526	(0.0224, 0.1297)	Beta (*α,β*)	9
LT (from HCC)	0.0800	(0.0760, 0.1196)	Beta (*α,β*)	9
Mortality risk ratios				
Non‐liver‐related mortality risk ratio: F0/F1	0.5117	(0.2700, 0.7500)	Beta (*α,β*)	9
Non‐liver related mortality risk ratio: F1/F2	0.4936	(0.2400, 0.7600)	Beta (*α,β*)	9
Non‐liver related mortality risk ratio: F2/F3	0.4931	(0.2400, 0.7500)	Beta (*α,β*)	9
Non‐liver related mortality risk ratio: F3/F4	0.4957	(0.2400, 0.7500)	Beta (*α,β*)	9
Non‐liver related mortality risk ratio: F4/DCC	0.2265	(0.1812, 0.2718)	Beta (*α,β*)	Calibrated
Liver‐related mortality risk ratio: F4/DCC	0.09	(0.072, 0.108)	Beta (*α,β*)	Calibrated
Risk ratio (MASH resolution)				
Resmetirom	2.54	(1.87, 3.55)	Gamma	11
Semaglutide	1.87	(1.6, 2.24)	Gamma	11
Tirzepatide	4.65	(2.31, 10.66)	Gamma	11
Risk ratio (fibrosis improvement)				
Resmetirom	1.64	(1.27, 2.2)	Gamma	11
Semaglutide	1.51	(1.23, 1.90)	Gamma	11
Tirzepatide	1.77	(1.17, 2.94)	Gamma	11
Annual Treatment costs ($)				
Resmetirom	59268.96	(47415.17, 71122.75)	Gamma	28
Semaglutide	16188.24	(12950.59, 19425.89)	Gamma	30
Tirzepatide	13036.44	(10429.15, 15643.73)	Gamma	31
Cost of doctor visit (per visit)	$129.07	($103.26, $154.89)	Gamma	18
Annual Health state costs ($)				
MASLD	0	N/A	Gamma	32
Non‐MASH F1	256.41	(205.13, 307.69)	Gamma	32
Non‐MASH F2	256.41	(205.13, 307.69)	Gamma	32
Non‐MASH F3	631.39	(505.11, 757.67)	Gamma	32
MASH F0	512.94	(410.35, 615.53)	Gamma	32
MASH F1	512.94	(410.35, 615.53)	Gamma	32
MASH F2	512.94	(410.35, 615.53)	Gamma	32
MASH F3	631.39	(505.11, 757.67)	Gamma	32
F4	22501.38	(18001.1, 27001.66)	Gamma	32
DCC	42439.09	(33951.27, 50926.91)	Gamma	32
HCC	110926.88	(88741.5, 133112.26)	Gamma	32
LT (year of)	480500.15	(384400.12, 576600.18)	Gamma	32
Post‐LT	15272.84	(12218.27, 18327.41)	Gamma	32
Utilities				
MASLD	1	(0.85, 1.00)	Beta (*α,β*)	15
Non‐MASH F1	1	(0.85, 1.00)	Beta (*α,β*)	15
Non‐MASH F2	1	(0.85, 1.00)	Beta (*α,β*)	15
Non‐MASH F3	0.84	(0.672, 1.000)	Beta (*α,β*)	15
MASH F0	0.85	(0.680, 1.000)	Beta (*α,β*)	15
MASH F1	0.772	(0.750, 0.795)	Beta (*α,β*)	19
MASH F2	0.671	(0.607, 0.736)	Beta (*α,β*)	19
MASH F3	0.605	(0.591, 0.620)	Beta (*α,β*)	19
MASH F4	0.500	(0.478, 0.523)	Beta (*α,β*)	19
DCC	0.251	(0.204, 0.299)	Beta (*α,β*)	19
LT (first year)	0.66	(0.49, 0.83)	Beta (*α,β*)	20
Post‐LT	0.73	(0.64, 0.82)	Beta (*α,β*)	20
HCC	0.5	(0.4, 0.6)	Beta (*α,β*)	20
Dis‐utility of Gastrointestinal AE	−0.02	(−0.024, −0.016)	Negative Beta	33
Gastrointestinal adverse events (%)				
Resmetirom	N/A			34
Semaglutide	23%	(18.4%, 27.6%)	Beta (*α,β*)	35
Tirzepatide	32%	(25.6%, 38.4%)	Beta (*α,β*)	7
First year treatment discontinuation rate (%)				
Resmetirom	4.3	(3.44%, 5.16%)	Beta (*α,β*)	34
Semaglutide	0	(0%, 3%)	Beta (*α,β*)	35
Tirzepatide	0	(0%, 3%)	Beta (*α,β*)	7
Discount rate (%)				
Discount rate	3	(1, 5)	Beta (*α,β*)	Assumption

### 2.2 MASH Natural History and Treatment Effects Modelling

Our model's natural history was based on a previously validated microsimulation of MASH progression [9]. This structure projects long‐term outcomes and incorporates the competing risks of liver‐related, non‐liver‐related and background mortalities. In adapting this structure, we initiated a hypothetical cohort at age 50 years, and we applied age‐specific mortality rates from the US life tables [10]. Our survival estimates and incidence of HCC and DCC were consistent with those reported in the validated MASH model (eFigures S1–S3 in Supporting Information); all validation targets were within the published confidence interval (CI) of the model for age 50 and MASH F2–F3 [9].

Bayesian network meta‐analysis (NMA) that anchored all therapies to placebo across randomized clinical trials was used to estimate the treatment effects for resmetirom, semaglutide and tirzepatide [11]. The FDA‐preferred histologic endpoints for treatment effects are: (1) one or more fibrosis stage improvements without worsening of MASH, and (2) MASH resolution without worsening of fibrosis [12]. The NMA reported risk ratios (RRs) for the two histologic endpoints. Each RR was converted into an annual treatment‐arm transition probability using the standard relationship between relative effects and transition probabilities described by Gidwani et al. [13] Fibrosis stage improvement was applied to modify transition risks between fibrosis stages, while MASH resolution was used to adjust transitions from MASH to non‐MASH health states. The two endpoints were modelled as separate, competing transitions with their RRs applied independently, avoiding double‐counting of treatment benefit.

A key treatment effect assumption (supported only by the resmetirom and semaglutide RCTs) was that treatment increased the likelihood of fibrosis improvement and MASH resolution while also reducing the probability of worsening by approximately 50%. This assumption served as the base‐case scenario, as it aligned with the available clinical trial evidence. However, because tirzepatide RCTs did not specify whether the benefits were derived from reductions in worsening or self‐transition (staying in the same fibrosis stage), we also considered an alternative conservative redistribution approach for tirzepatide. The full derivation of treatment‐arm transition probabilities, including the equations, redistribution rules, and a worked numerical example, is provided in the eAppendix in supplemental materials.

In our model, the treatment efficacy is held constant during active treatment, with no waning of effect assumed. We adopted a conservative modelling structure for fibrosis improvement by allowing at most one fibrosis stage transition per cycle. Patients who discontinue treatment transition between states according to standard‐of‐care probabilities (i.e., resumption of natural disease progression from the stage achieved during treatment). This assumption is supported by recently presented extension data in which patients who paused resmetirom therapy experienced evidence of disease progression by biomarker and imaging measures, with improvements restored upon treatment resumption [14]. Equivalent data for semaglutide and tirzepatide in MASH‐specific populations are not yet available, so we assumed the same post‐discontinuation behaviour applies to all three agents.

### 2.3 Costs and Utilities

Health care costs for each disease state were derived from a previously published economic evaluation [15] and adjusted to USD 2024 using the US Consumer Price Index–Medical Care Component [16] from the Bureau of Labour Statistics [17]. All drug prices used in the model reflect wholesale acquisition cost (WAC) values as listed by the manufacturers. Because tirzepatide is not yet FDA‐approved for MASH, no MASH‐specific list price exists. Therefore, we used Zepbound's WAC as the base‐case price input in our model, because the obesity indication is clinically and commercially more analogous to the MASH F2–F3 population. We additionally tested a range of prices in a one‐way sensitivity analysis. Adverse event modelling was restricted to gastrointestinal symptoms (nausea, diarrhoea, constipation and vomiting), which were the most frequently reported treatment‐emergent events in the trials of semaglutide and tirzepatide. The cost of one additional gastroenterology visit was included for patients receiving semaglutide and tirzepatide to account for potential management of gastrointestinal adverse events, which was derived from the Centres for Medicare and Medicaid Services (CMS) [18].

Stage‐specific utilities for MASH and fibrosis F1 through decompensated cirrhosis were taken from a primary EQ‐5D‐5L and discrete choice experiment elicitation study [19]. For hepatocellular carcinoma (HCC), the liver transplant (LT) procedure year, and post‐transplant states, we used previously published utility values from the Institute for Clinical and Economic Review's evaluation [20]. For MASLD and non‐MASH F1–F3 states, utility values are consistent with the approach taken in the recently published cost‐effectiveness analysis [15]. Costs and quality‐adjusted life‐years were accrued at the end of each annual cycle.

### 2.4 Economic and Sensitivity Analyses

The primary outcome was the incremental cost‐effectiveness ratio (ICER), which was assessed using a willingness‐to‐pay (WTP) threshold of $100 000 per QALY [21, 22]. Our base‐case analysis compared each pharmacotherapy individually against the SoC. This approach was deliberately chosen because the treatment effects were estimated using placebo‐anchored NMA.

To assess the model robustness, we conducted one‐way sensitivity analyses of the key input parameters. Parameter ranges were obtained from the published literature, with treatment‐related parameters varied according to the credible intervals (Crl) reported in the network meta‐analysis; other parameters were ranged by 20% for upper and lower values. In addition, we have conducted a probabilistic sensitivity analysis (PSA), in which parameters were sampled from their assumed probability distributions. Costs and utilities were varied by ±20% of their base‐case values or within the confidence intervals (CI) if reported. For the PSA, the cost parameters followed a gamma distribution. Other parameters in the range of [0, 1] were modelled using a beta distribution with disutilities applied as the negative of the sampled beta value to reduce the baseline state utility.

Additional sensitivity analyses have been conducted to evaluate the robustness of the results by varying the risk ratios of the endpoints and conservative utility values. In addition, to address uncertainty in long‐term treatment persistence, we conducted two scenario analyses: a one‐way scan of annual per‐cycle discontinuation rates from 0% to 50% for each pharmacotherapy, and a finite treatment‐duration analysis with fixed treatment caps of 2, 5, and 10 years. In both analyses, patients who discontinued or reached the treatment cap ceased drug costs but continued to incur health‐state management costs and reverted to SoC transition probabilities. To inform pricing strategies and payer decisions, we conducted threshold pricing analyses for each pharmacotherapy versus SoC at 3 U.S. willingness‐to‐pay thresholds.

## 3 Results

### 3.1 Base‐Case Economic Outcomes

The results of the base‐case analysis, comparing each pharmacotherapy individually against SoC are presented in Table 2. All three pharmacotherapy strategies provided meaningful health gains compared with SoC over a lifetime horizon but demonstrated vastly different economic values. Compared to SoC, resmetirom increased the QALYs by 3.47, but at a high incremental cost of $948 743, resulting in an ICER of $273 445 per QALY. Semaglutide increased QALYs by 3.04 at an incremental cost of $243 094 (ICER: $80 076 per QALY). Tirzepatide produced the largest QALY gain of 4.46 at the lowest incremental cost ($190 270), yielding an ICER of $42 705 per QALY. Among the three pharmacotherapies evaluated against SoC, tirzepatide had the largest QALY gain and the lowest incremental cost. However, SoC remained the least costly overall strategy. At the conventional WTP threshold of $100 K per QALY, tirzepatide and semaglutide were cost‐effective, whereas resmetirom exceeded this benchmark by more than two‐fold.

**TABLE 2:** Base case results of pharmacotherapy versus standard of care. This table summarises the total costs, total QALYs, incremental costs, incremental QALYs, and ICERs for each pharmacotherapy compared to the standard of care (SoC). Incremental outcomes were calculated relative to SoC, and ICERs were reported as cost per QALY gained. ICER, incremental cost‐effectiveness ratio; QALY, quality‐adjusted life‐year; SoC, standard of care; WTP, willingness‐to‐pay.

Treatment	Cost ($)	QALYs	Incremental cost ($)	Incremental QALYs	ICER ($/QALYs)
SoC	54947.96	11.98	—	—	—
Resmetirom	1 003 691	15.45	948743.05	3.47	273 445
Semaglutide	298042.59	15.02	243094.64	3.04	80 076
Tirzepatide	245218.87	16.44	190270.92	4.46	42 705

*Note:* Cost‐effectiveness is defined by an ICER below the conventional U.S. willingness‐to‐pay (WTP) threshold of $100 000 per QALY. ICERs reflect individual comparisons of each pharmacotherapy versus SoC.

The results of the 10‐year outcome and additional scenario analysis (normalisation scenario) are provided in the Supporting Information (eTable S1 and S2 in Supporting Information). Over a 10‐year period, resmetirom had an ICER of $418 006 per QALY, semaglutide had an ICER of $137 422, and tirzepatide had an ICER of $54 405 per QALY.

For threshold pricing analysis, at a $100 000/QALY threshold, the maximum annual cost‐effective price would be approximately $23 007 for resmetirom, a 61% reduction from its current WAC of $59 269. Semaglutide and tirzepatide already meet this threshold at their current WAC. At the stricter $50 000/QALY threshold, all three agents would require price reductions of approximately 79%, 33%, and 14%, respectively. At the more permissive $150 000/QALY threshold, only resmetirom requires a price reduction of approximately 44%. Full results are shown in eTable S3 in the Supporting Information.

### 3.2 Sensitivity Analyses

**FIGURE 2:**
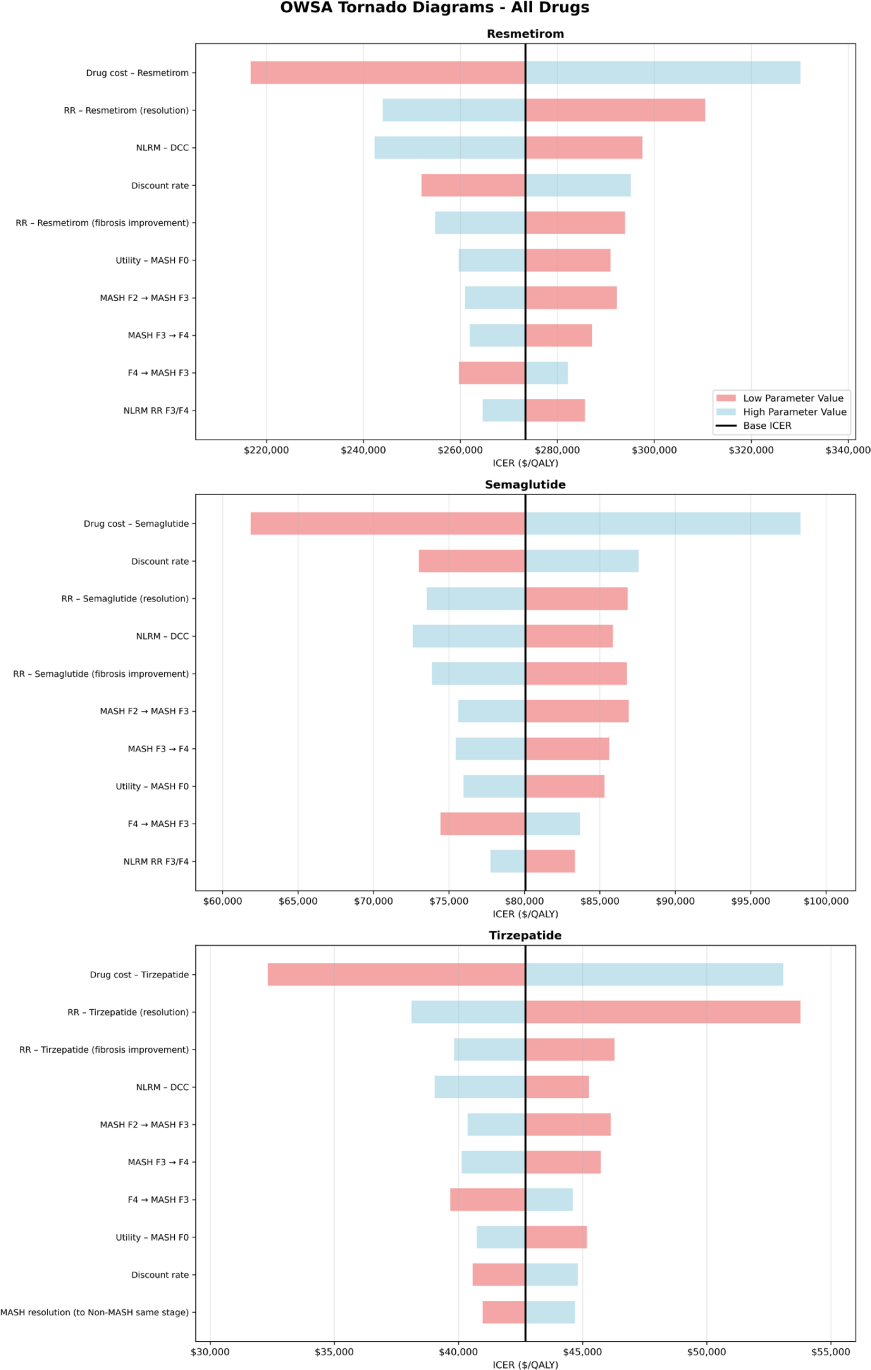
One‐way sensitivity analysis for three strategies over the lifetime horizon. This figure presents tornado diagrams showing the impact of parameter uncertainty on the ICERs for resmetirom, semaglutide and tirzepatide, each compared with the standard of care. For each strategy, the parameters were varied across their plausible ranges to illustrate how individual inputs influenced the cost‐effectiveness results. Bars represent ICERs under low and high parameter values relative to the base‐case reference line. RR, risk ratio; ICER, incremental cost‐effectiveness ratio; NLRM, non‐liver‐related mortality; QALY, quality‐adjusted life years.

One‐way sensitivity analyses consistently showed that drug cost was the most influential input parameter for the cost‐effectiveness of all three pharmacotherapies (Figure 2). The next most influential parameters common to all strategies included non‐liver‐related mortality (NLRM) at DCC and fibrosis improvement/resolution risk ratios. Among the three agents, tirzepatide carries the lowest annual list price and produced the largest QALY gain in the base case, which together drive its favourable ICER. Because drug acquisition cost dominates the total incremental cost in all three pharmacotherapy arms, agents with lower per‐year prices will tend to produce more favourable ICERs even when their absolute clinical efficacy is comparable. The economic ordering of the three pharmacotherapies versus SoC is therefore as much a function of pricing differentials as of efficacy differences.

In a sensitivity analysis reducing the utility of MASLD, non‐MASH F1 and non‐MASH F2 states from 1.0 to 0.85, ICERs versus SoC increased for all three pharmacotherapies. Tirzepatide remained cost‐effective, semaglutide remained at the boundary of conventional willingness‐to‐pay thresholds, and resmetirom remained uneconomical. Full results are presented in Supporting Information eTable S4.

Two‐way sensitivity analysis on the NMA‐derived treatment‐effect risk ratios demonstrated that cost‐effectiveness conclusions for semaglutide and tirzepatide were robust to joint variation across the 95% CrI, below the $100 000/QALY threshold. Resmetirom ICERs exceeded the threshold across all four boundary combinations (eTable S5).

To address uncertainty in long‐term treatment persistence, we conducted a one‐way sensitivity analysis of annual per‐cycle discontinuation rates from 0% to 50% for all three agents (eFigure S4). ICERs declined as the yearly discontinuation rate increased for all agents. For resmetirom, the ICER declined to less than the $100 000/QALY threshold at approximately 37% annual discontinuation. Semaglutide and tirzepatide remained below the $100 000/QALY threshold across the entire range tested. For a fixed treatment‐duration analysis, caps of 2, 5 and 10 years produced ICERs that declined with shorter cap durations across all three pharmacotherapies (eFigure S5). For semaglutide and tirzepatide, ICERs remained below the $100 000/QALY threshold at every cap duration. For resmetirom, the ICER crossed the $100 000/QALY threshold only under a 2‐year cap. Because MASH is a chronic progressive disease for which all three therapies are intended for long‐term use, this finding does not support short‐course treatment in clinical practice but rather highlights that resmetirom's unfavourable lifetime ICER is driven by cumulative drug acquisition costs.

**FIGURE 3:**
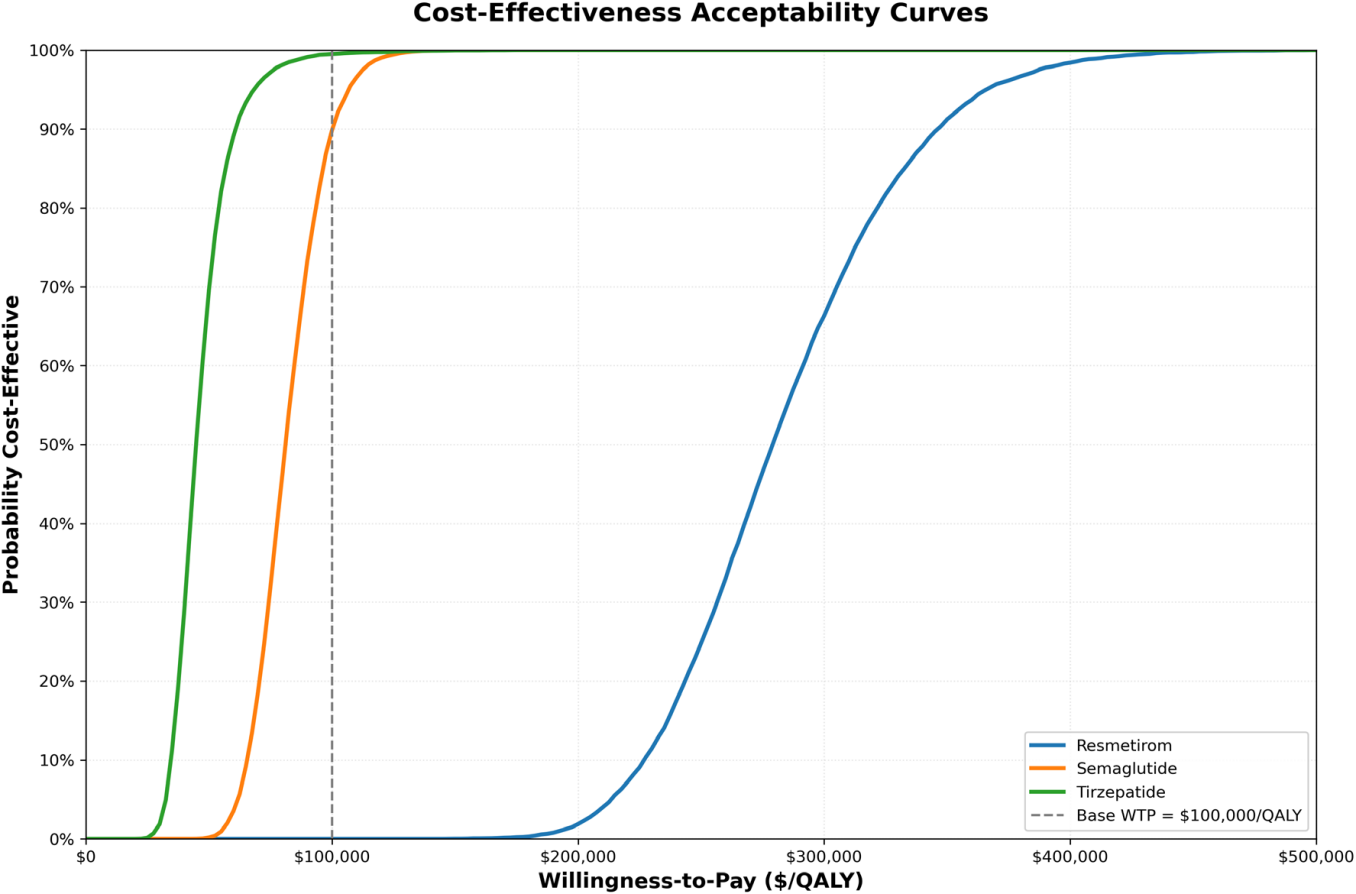
Cost‐effectiveness acceptability curves for pharmacotherapies for MASH over a lifetime horizon. This figure displays the probability that resmetirom, semaglutide and tirzepatide are cost‐effective across a range of WTP thresholds. Curves represent the proportion of probabilistic sensitivity analysis simulations in which each treatment is cost‐effective relative to the standard of care at a given willingness‐to‐pay (WTP) threshold. The dashed vertical line indicates the $100 000 per QALY threshold commonly used in U.S. cost‐effectiveness analyses. QALY, quality‐adjusted life year; WTP, willingness‐to‐pay.

**FIGURE 4:**
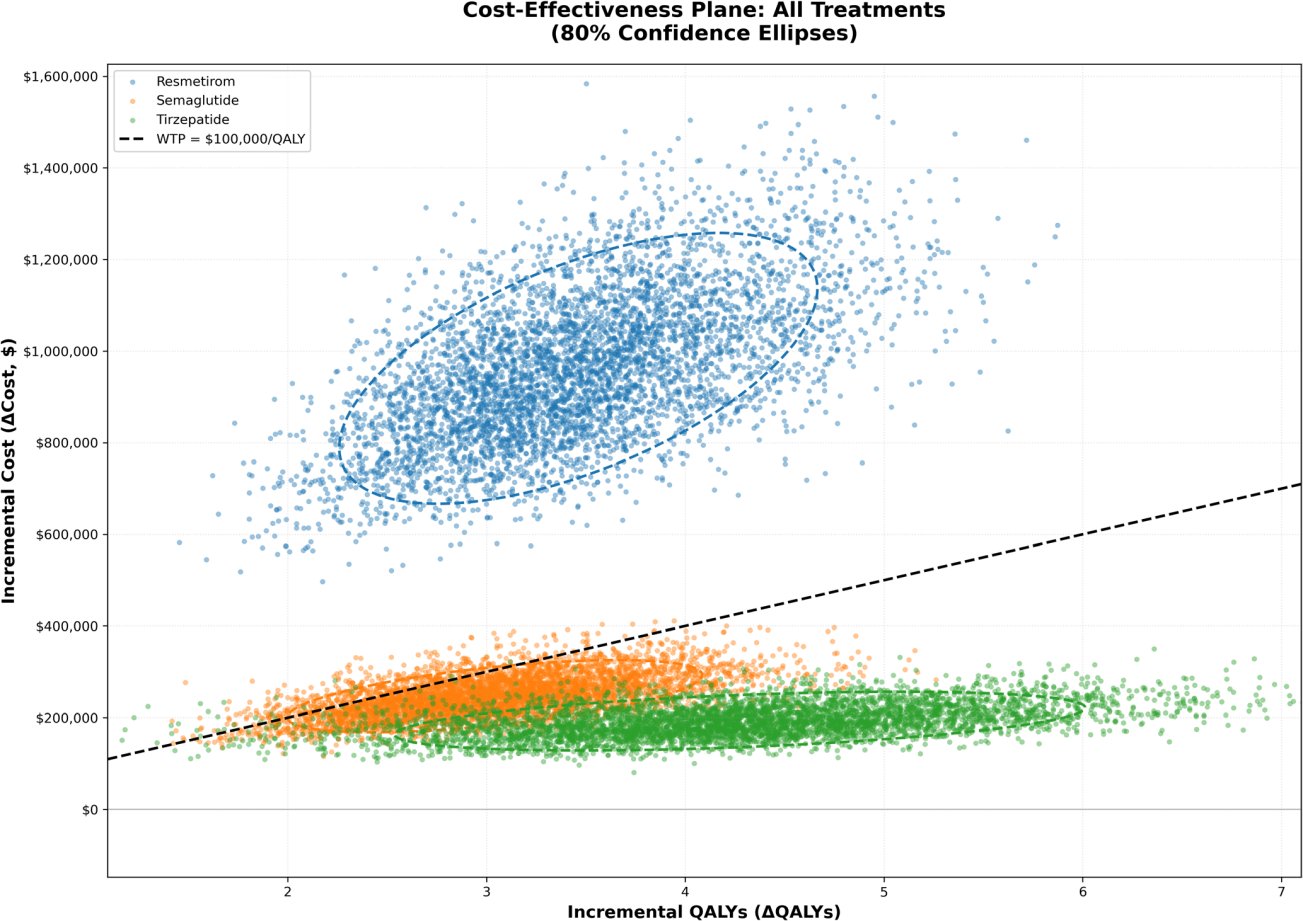
Cost‐effectiveness plane for pharmacotherapies for MASH over a lifetime horizon. This figure illustrates the scatter of incremental costs and incremental QALYs generated from probabilistic sensitivity analysis simulations for resmetirom, semaglutide and tirzepatide compared to the standard of care. Each point represents one simulation iteration. The dashed line represents the $100 000 per QALY willingness‐to‐pay (WTP) threshold. QALY, quality‐adjusted life year; WTP, willingness‐to‐pay.

Probabilistic sensitivity analyses (PSA) were conducted for all strategies by running 10 000 simulations per strategy (Figures 3 and 4). At the $100 000/QALY WTP threshold, the probability of cost‐effectiveness was 0% for resmetirom, 89.8% for semaglutide, and 99.5% for tirzepatide. Figure 3 shows the cost‐effectiveness acceptability curves for each pharmacotherapy versus SoC across a range of WTP thresholds. Taken together, tirzepatide was the most robustly cost‐effective compared with SoC; semaglutide has a high probability (89.8%) of being cost‐effective, and resmetirom is robustly unlikely (0% probability) to be considered an acceptable economic value under current pricing.

## 4 Discussion

In this economic evaluation, pharmacologic therapy for MASH with stage F2–F3 fibrosis produced meaningful health gains compared with the standard of care. However, only tirzepatide and semaglutide met conventional U.S. willingness‐to‐pay thresholds of $100 000 per QALY, whereas resmetirom did not, with an ICER more than twice this amount over the lifetime horizon. Drug cost was the dominant determinant of cost‐effectiveness across all scenarios, underscoring the central role of pricing in shaping the value of new MASH therapies.

Our results align with early value‐based pricing analyses of resmetirom and extend prior work by incorporating recently approved incretin therapies and using MASH stage‐specific utility values. In a recent analysis, Le et al. estimated an incremental cost‐effectiveness ratio of approximately $100 000 per QALY for resmetirom compared with standard of care and identified value‐based annual prices $8132 to $28 181, depending on different treatment discontinuation assumptions [15]. Our analysis expands on prior work in several methodological aspects. We evaluated three pharmacotherapies (resmetirom, semaglutide and tirzepatide) within a unified modelling framework, with each agent compared individually against SoC using a placebo‐anchored Bayesian network meta‐analysis to estimate treatment effects. The model incorporated both FDA‐preferred histologic endpoints of MASH resolution and fibrosis improvement for all three agents. The favourable economic value of tirzepatide compared to SoC, achieving the lowest ICER and 99.5% probability of cost‐effectiveness in the probabilistic sensitivity analysis, suggests that incretin‐based therapies may serve as a useful reference point for evaluating future MASH pharmacotherapies.

The introduction of pharmacologic treatments for MASH represents an inflection point for liver and metabolic disease care. However, the high price of the first‐approved agent, resmetirom (annual cost: $59269), poses an immediate policy challenge. Treating only the eligible U.S. adults with biopsy‐confirmed MASH F2‐F3 could require annual spending exceeding $10 billion. Similar to the experience with curative therapies for hepatitis C, uncoordinated adoption could strain payer budgets, delay coverage and widen disparities in access [23, 24, 25].

Thus, to translate pharmacologic innovation into population‐level benefit, pricing and reimbursement strategies must evolve in parallel. Outcomes‐based contracts, indication‐specific pricing, and volume‐linked rebates could align incentives between manufacturers and payers while preserving affordability. MASH disproportionately affects populations with obesity, type 2 diabetes, and those of lower socioeconomic status who are often underinsured. The current price disparity among agents, where the most cost‐effective options are significantly cheaper, indicates that uniform pricing will severely limit access and exacerbate metabolic health inequities.

Our findings should be interpreted in the context of several limitations. First and most importantly, our base‐case model was designed to be a conservative, liver‐centric analysis, and therefore it does not incorporate the proven cardiovascular and all‐cause mortality and weight‐loss benefits of GLP‐1 receptor agonists. This is a significant omission, as cardiovascular events represent the leading cause of mortality in the MASH population and act as a critical competing risk for liver‐related outcomes. By excluding these benefits, our model substantially underestimates the true, holistic value of semaglutide and tirzepatide. The ICERs presented for tirzepatide ($42 705/QALY) and semaglutide ($80 076/QALY) should therefore be interpreted as conservative estimates of their general cost‐effectiveness. The tirzepatide analysis carries additional uncertainty. Its efficacy utilised in the NMA is from a Phase 2 trial, with substantially wider CrI than the FDA‐approved agents; therefore, findings should be interpreted with caution until Phase 3 confirmation and MASH‐indication pricing are available.

Second, we reported ICERs for each therapy compared with the standard of care rather than performing a sequential incremental analysis against an ‘efficiency frontier’. This approach was chosen because treatment effects were estimated from a placebo‐anchored network meta‐analysis. In the absence of head‐to‐head trials, performing sequential comparisons on NMA‐derived data could introduce a risk of bias by relying on transitivity assumptions [26]. Third, our SoC arm represents MASH natural history under management without active MASH‐targeted pharmacotherapy. The underlying transition probabilities and mortality estimates derive from cohorts enrolled and followed between approximately 2004 and 2018, predating the widespread use of GLP‐1 receptor agonists. We acknowledge that liraglutide, the only GLP‐1 RA approved for type 2 diabetes prior to 2017, may have been used as background antidiabetic therapy in some patients within these source cohorts. However, in placebo‐anchored NMA structure, the treatment‐effect estimates for all agents used in this study were obtained by pooling trials including other metabolically active comparators (e.g., obeticholic acid, liraglutide and pioglitazone). The risk ratios are therefore anchored against a placebo that already reflects mixed background metabolic care. Additionally, a recent sub‐analysis of the MAESTRO‐NASH trial reported that 13%–17% of patients with type 2 diabetes were on stable GLP‐1 receptor agonist or SGLT2 inhibitor therapy at baseline; in that subgroup, rates of MASH resolution and fibrosis improvement were similar to those of patients not receiving these therapies, providing direct evidence that background metabolic therapy did not meaningfully alter MASH histologic outcomes in F2–F3 patients using resmetirom [27]. Our sensitivity analyses sampled treatment‐effect estimates across the wide CrI reported in the source NMA, providing robustness against this residual uncertainty.

Fourth, our analysis used a cohort‐based Markov model adapted from a previously validated microsimulation. While this model did not incorporate sex‐specific background mortality, our validation against the original microsimulation demonstrated that sex‐related differences were not significant. Fifth, adverse event modelling was restricted to gastrointestinal symptoms, which are the dominant treatment‐related adverse events for the semaglutide and tirzepatide classes of medication in the source trials. We did not separately incorporate the costs or disutility of serious adverse events such as pancreatitis, gallbladder events, severe dehydration, or hospitalisation for any reason, because the published randomized trials did not demonstrate clinically meaningful differences between active treatment and placebo arms. Real‐world data may show severe GI complications not captured in trials, so ICERs for semaglutide and tirzepatide should be interpreted as lower‐bound estimates with respect to AE burden. Finally, our analysis used the wholesale acquisition cost (WAC) for all used agents. For resmetirom, the cost was based on data from Madrigal Pharmaceuticals for the state of Vermont [28]. We used the most conservative WAC plus 20%. Even with a pricing sensitivity analysis, it cannot achieve an ICER value at or below the WTP threshold. For semaglutide and tirzepatide, we also used the WAC published on the manufacturers' websites. The Medicare Drug Price Negotiation Program has proposed a 70% price reduction by 2027, which will significantly influence the cost‐effectiveness, availability, and coverage of these drugs for Medicare beneficiaries [29].

## 5 Conclusion

At current U.S. prices, tirzepatide ($42 705/QALY) and semaglutide ($80 076/QALY) appear cost‐effective compared with SoC for patients with MASH and stage F2–F3 fibrosis, whereas resmetirom does not meet acceptable threshold values. The favourable economic value of tirzepatide compared with SoC, while requiring confirmation through Phase 3 trials and head‐to‐head comparisons, suggests that incretin‐based therapies may represent a useful reference point for evaluating the value of future MASH pharmacotherapies. Drug pricing, not clinical efficacy, will ultimately determine the real‐world impact of pharmacologic therapy and the growing burden of MASH. Aligning clinical innovation with value‐based pricing and equitable access is critical to ensure that the advent of effective therapy translates into population‐level health gains rather than widening disparities in metabolic liver disease.

## Author Contributions

**Ali A. Abdeen:** conceptualization, methodology, software development, formal analysis, data collection and writing of the original draft. **Aanan Biswas:** software development, formal analysis and validation. **Turgay Ayer:** conceptualization, supervision and resources. **Chase J. Wehrle:** writing – review and editing. **Sobia N. Laique:** methodology, validation and writing – review and editing. **Ali Aminian:** methodology, validation and writing – review and editing.

## Funding

The authors have nothing to report.

## Conflicts of Interest

S. L. serves as consultant for Novo Nordisk and Madrigal Pharmaceuticals. A, Aminian reported receiving research grants from Amgen, Ethicon, and Medtronic. He serves as a consultant for Amylyx, Eli Lilly, Ethicon, and Medtronic. Other authors declared no conflicts of interest. C Wehrle is a consultant for HistoSonics Inc.

## Supporting information


**eFigure S1:** Ten‐year survival for MASH F2 and F3.
**eFigure S2:** Ten‐year cumulative incidence rate of hepatocellular carcinoma for MASH F2 and F3.
**eFigure S3:** Ten‐year cumulative incidence rate of decompensated cirrhosis for MASH F2 and F3.
**eFigure S4:** One‐way sensitivity of incremental cost‐effectiveness ratio to annual treatment discontinuation rate for resmetirom, semaglutide and tirzepatide versus standard of care.
**eFigure S5:** ICER sensitivity to treatment‐duration cap.
**eTable S1:** Base‐case results for 10‐year time horizon.
**eTable S2:** Base‐case results for the normalisation scenario over the life‐time horizon.
**eTable S3:** Maximum annual drug price at which each pharmacotherapy would be cost‐effective versus standard of care at three willingness‐to‐pay thresholds
**eTable S4:** Base‐case results for a conservative early MASLD and Non‐MASH (F1–F2) states.
**eTable S5:** Two‐way sensitivity analysis of MASH resolution and fibrosis improvement risk ratios.

**Data S1:** Supporting Information.

## Data Availability

The data that support the findings of this study are openly available in the published sources cited within the manuscript and summarized in Table 1 and reference numbers [9, 10, 11], [15], [17, 18, 19, 20], [28], [30, 31, 32, 33, 34, 35].
