# Supplementary material for: Cost‐Effectiveness of Pharmacologic Therapies for Metabolic Dysfunction–Associated Steatohepatitis With Significant Fibrosis in the United States
Source: Diabetes Obes Metab. 2026 Jun 25;28(9):8378–89. doi: 10.1111/dom.71020 (PMC13449014; doi:10.1111/dom.71020)
Supplement: Supplementary file 2 [file DOM-28-8378-s002.docx]

**Supplemental materials**

Cost-Effectiveness of Pharmacologic Therapies for Metabolic Dysfunction–Associated Steatohepatitis with Significant Fibrosis in the United States

eFigure 1: Ten-year survival for MASH F2 and F3. (Page 2)

eFigure 2: Ten-year cumulative incidence rate of hepatocellular carcinoma for MASH F2 and F3. (Page 3)

eFigure 3: Ten-year cumulative incidence rate of decompensated cirrhosis for MASH F2 and F3. (Page 4)

# eAppendix: Methodology for Deriving Annual Transition Probabilities for Treatment Arms (Page 5-8)

eTable 1: Base-case results for 10-year time horizon. (Page 9)

eTable 2: Base-case results for the time horizon for the normalization scenario over the life-time horizon. (Page 9)

eTable 3: Maximum annual drug price at which each pharmacotherapy would be cost-effective versus standard of care at three willingness-to-pay thresholds (Page 9)

eTable 4: Base-case results for a conservative early MASLD and Non-MASH (F1-F2) states. (Page 10)

eTable 5: Two-way sensitivity analysis of MASH resolution and fibrosis improvement risk ratios. (Page 11)

eFigure 4: One-way sensitivity of incremental cost-effectiveness ratio to annual treatment discontinuation rate for resmetirom, semaglutide, and tirzepatide versus standard of care. (Page 12)

eFigure 5: ICER sensitivity to treatment-duration cap. (Page 13)

References (Page 14)

eFigure 1: Ten-year survival for MASH F2 and F3.

eFigure 2: Ten-year cumulative incidence rate of hepatocellular carcinoma for MASH F2 and F3.

eFigure 3: Ten-year cumulative incidence rate of decompensated cirrhosis for MASH F2 and F3.

# eAppendix : Methodology for Deriving Annual Transition Probabilities for Treatment Arms

The standard of care (SoC) arm of our model, including the overall structure and the annual transition probabilities, is based directly on the natural history model published by Chhatwal et al.^1^ The treatment arms of our model follow the same underlying structure—they feature the same disease states and possible transitions—but use different annual transition probabilities. These probabilities were derived from risk ratios (RRs) estimated by Souza et al. through a Bayesian network meta-analysis of trial-level histological endpoints.^2^ Dias et al. corroborate that combining relative treatment effects with a natural history baseline to model disease progression under treatment is a standard approach in the CEA literature.^3^

A risk ratio equals the probability, $p_{1}$, of an event in the exposed group divided by the probability, $p_{0}$, of the same event in the unexposed group (Gidwani et al.).^4^ In Souza et al., the exposed group consists of patients on a particular treatment, and the unexposed group consists of patients on the standard of care.^2^

$$RR=\frac{p_{1}}{p_{0}} \left( 1 \right)$$

These risk ratios describe the comparative benefit of taking medication over adhering to the standard of care. The following equation is used to calculate a transition probability from a RR (Gidwani et al.)^4^:

$$p_{1}=RR\times p_{0} \left( 2 \right)$$

However, Gidwani et al. include an important caveat: when $p_{0}$ and $RR$ come from different sources, they must be derived from sufficiently similar populations for the resulting estimate of $p_{1}$ to be valid and applicable. The RRs reported in Souza et al. are synthesized from randomized controlled trials with patients who had biopsy-proven MASH. The endpoints in these trials were fibrosis improvement without MASH worsening and MASH resolution without fibrosis worsening. On the other hand, the natural history model published by Chhatwal et al., which supplied the SOC transition probabilities, or $p_{0}$, also relied on randomized, placebo-controlled clinical trials with the same underlying histological endpoints (fibrosis improvement without MASH worsening and MASH resolution without worsening fibrosis). Therefore, the condition laid out by Gidwani et al. is met.

Souza et al. provide two RRs for every treatment, one describing the relative benefit of treatment with regard to fibrosis improvement and the other with regard to MASH resolution.

Applying (1), the RRs can be written as the following:

$$RR_{\mathrm{fibrosis}}=\frac{p_{i\to j,fibrosis}^{\mathrm{treatment}}}{p_{i\to j,fibrosis}^{\mathrm{SOC}}} \left( 3 \right)$$

$$RR_{\mathrm{MASH}}=\frac{p_{\mathrm{MASH}}^{\mathrm{treatment}}}{p_{\mathrm{MASH}}^{\mathrm{SOC}}} \left( 4 \right)$$

Here, $p_{i\to j,fibrosis}^{\mathrm{treatment}}$ and $p_{i\to j,fibrosis}^{\mathrm{SOC}}$ denote the probabilities of fibrosis improvement from state $i$ to state $j$ under treatment and SOC, respectively. Likewise, $p_{\mathrm{MASH}}^{\mathrm{treatment}}$ and $p_{\mathrm{MASH}}^{\mathrm{SOC}}$ denote the probabilities of MASH resolution under treatment and SOC, respectively. Note that the state is not needed in the second definition, since a constant annual probability of MASH resolution is assumed across NASH F1-F3 (Chhatwal et al.).

Applying (2), we get the following:

$$p_{i\to j,fibrosis}^{\mathrm{treatment}}=RR_{\mathrm{fibrosis}}\times p_{i\to j,fibrosis}^{\mathrm{SOC}} \left( 5 \right)$$

$$p_{\mathrm{MASH}}^{\mathrm{treatment}}=RR_{\mathrm{MASH}}\times p_{\mathrm{MASH}}^{\mathrm{SOC}} \left( 6 \right)$$

To avoid double counting treatment benefit, we made the conservative assumption, consistent with the natural history model published by Chhatwal et al., that patients cannot experience both fibrosis improvement and MASH resolution in the same cycle. Fibrosis improvement and MASH resolution were therefore modeled as separate, competing transitions, allowing their respective RRs to be applied independently.

For a concrete application of these definitions, consider a patient in the MASH-F2 state on the medication Resmetirom. The possible one-year transitions for this patient are to non-MASH F2 (MASH resolution), MASH F2 (self-transition), MASH F1 (fibrosis improvement), and MASH F3 (fibrosis worsening). In the SOC arm, the respective probabilities of these transitions are 0.08, 0.6977, 0.0810, and 0.1413 (note that all probabilities in this example are reported prior to scaling by the survival probability for simplicity). For Resmetirom, $RR_{\mathrm{fibrosis}}=1.64$ and $RR_{\mathrm{MASH}}=2.54$. Using (5) and (6), we obtain:

$$p_{MASH F2\to MASH F1}^{\mathrm{Resmetirom}}=RR_{\mathrm{fibrosis}}\times p_{MASH F2\to MASH F1}^{\mathrm{SOC}}=1.64\times0.0810=0.13284.$$

$$p_{MASH F2\to non\text{-}MASH F2}^{\mathrm{Resmetirom}}=RR_{\mathrm{MASH}}\times p_{\mathrm{MASH}}^{\mathrm{SOC}}=2.54\times0.08=0.2032.$$

However, these formulas define only a subset of the treatment-arm transition probabilities, leaving open how to redistribute the remaining transition probabilities. These remaining probabilities cannot simply equal their SOC counterparts, since increasing the fibrosis improvement and MASH resolution probabilities causes the row sum to exceed 1.

Absent direct guidance from the literature on this redistribution, we modeled two different approaches. Under the first approach, which served as the base case, we reduced the probability of fibrosis worsening to compensate for the increase in fibrosis improvement and MASH resolution probabilities. This assumption was motivated by data from Resmetirom’s RCT: 34% of patients on the SOC experienced worsening, 51% experienced no change, and 15% experienced improvement; among patients on 80 mg of Resmetirom, 18% experienced worsening, 51% experienced no change, and 31% experienced improvement. In the trial, the increase in proportion of patients with fibrosis improvement came entirely from a reduction in proportion of patients with fibrosis worsening.

However, an unintended consequence of this approach is that if a RR is very large, the worsening probability will reach 0 to fully fund the increased probability of improvement, possibly overexaggerating the treatment benefit. (Note that “improvement” entails both fibrosis improvement and MASH resolution, and “worsening” entails both fibrosis worsening and MASH relapse, if applicable.) Hence, a floor was incorporated, below which the worsening probability could not drop. We considered that 50% of the SOC worsening probability consistent with the worsening rates observed in the treatment arms of the resmetirom and semaglutide Phase 3 trials. A high floor delivers a conservative estimate of the treatment benefit, since the treatment worsening probability cannot be much lower than the SOC worsening probability, whereas a low floor delivers more optimistic outcomes.

Continuing with the example, the worsening probability, $p_{MASH F2\to MASH F3}^{\mathrm{SOC}}=0.1413$, must be reduced by the same amount that the fibrosis improvement and MASH resolution probabilities were increased:

$$\left[ p_{MASH F2\to MASH F1}^{\mathrm{Resmetirom}}-p_{MASH F2\to MASH F1}^{\mathrm{SOC}} \right]+\left[ p_{MASH F2\to non\text{-}MASH F2}^{\mathrm{Resmetirom}}-p_{MASH F2\to non\text{-}MASH F2}^{\mathrm{SOC}} \right]=0.17504.$$

(Note that if multiple transitions are classified as worsening, the ratio of their SOC transition probabilities is maintained after the reduction.)

However, with a floor of 50%, the lowest possible value $p_{MASH F2\to MASH F3}^{\mathrm{Resmetirom}}$ can take on is:

$$p_{MASH F2\to MASH F3}^{\mathrm{Resmetirom}}=0.5\times p_{MASH F2\to MASH F3}^{\mathrm{SOC}}=0.07065.$$

The resulting transition probabilities are 0.2032, 0.6977, 0.13284, and 0.07065, which do not sum to 1.

In such cases, where reducing the worsening probability is not enough, the self-transition probability is reduced to fund the rest of the increase in improvement probability. The following are the annual transition probabilities for Resmetirom from the MASH F2 state (prior to scaling by the survival probability):

$$p_{MASH F2\to non\text{-}MASH F2}^{\mathrm{Resmetirom}}=0.2032$$

$$p_{MASH F2\to MASH F2}^{\mathrm{Resmetirom}}=0.6977-\left[ \left( 0.2032+0.6977+0.13284+0.07065 \right)-1 \right]=0.593$$

$$p_{MASH F2\to MASH F1}^{\mathrm{Resmetirom}}=0.13284$$

$$p_{MASH F2\to MASH F3}^{\mathrm{Resmetirom}}=0.07065$$

Under the second approach, the transition probabilities are directly normalized so that they sum to 1. In other words, after the SOC fibrosis improvement probability and SOC MASH resolution probability are multiplied by $RR_{\mathrm{fibrosis}}$ and $RR_{\mathrm{MASH}}$, respectively, each transition probability is divided by the sum of the probabilities:

$$p_{MASH F2\to non\text{-}MASH F2}^{\mathrm{Resmetirom}}=\frac{0.2032}{0.2032+0.6977+0.13284+0.1413}=0.173$$

$$p_{MASH F2\to MASH F2}^{\mathrm{Resmetirom}}=\frac{0.6977}{0.2032+0.6977+0.13284+0.1413}=0.594$$

$$p_{MASH F2\to MASH F1}^{\mathrm{Resmetirom}}=\frac{0.13284}{0.2032+0.6977+0.13284+0.1413}=0.113$$

$$p_{MASH F2\to MASH F3}^{\mathrm{Resmetirom}}=\frac{0.1413}{0.2032+0.6977+0.13284+0.1413}=0.120$$

This approach is more conservative compared to the base case approach because the worsening probability is not explicitly reduced; additionally, the fibrosis improvement and MASH resolution probabilities are scaled down by some factor due to normalization. As a result, the ratios given by the model’s actual transition probabilities are smaller than the RRs from Souza et al., suggesting that this method potentially understates the treatment benefit. By comparison, an advantage of the base case approach is that:

$$\frac{p_{i\to j,fibrosis}^{\mathrm{treatment}}}{p_{i\to j,fibrosis}^{\mathrm{SOC}}}\approx RR_{\mathrm{fibrosis}}$$

$$\frac{p_{\mathrm{MASH}}^{\mathrm{treatment}}}{p_{\mathrm{MASH}}^{\mathrm{SOC}}}\approx RR_{\mathrm{MASH}}$$

Ultimately, a scenario analysis was necessary to assess the impact of this structural uncertainty on the model’s results as shown in eTable 2. Three additional methodological assumptions warrant explicit acknowledgment. First, the Souza et al. RRs are reported as posterior medians from a Bayesian random-effects NMA; we used these as the central estimates in the base case and sampled across their full 95% CI in probabilistic and two-way sensitivity analyses. Second, the Souza et al. RRs derive from trial-level histologic endpoints assessed predominantly at 48–52 weeks follow-up. Because trial follow-up duration approximates the one-year cycle length of our model, we applied these RRs as direct multipliers to annual transition probabilities without further temporal adjustment; the validity of this mapping rests on the assumption that 52-week event probabilities can be approximated as annualized transition probabilities in a one-year-cycle Markov framework. Third, RR_fibrosis and RR_MASH from Souza et al. are pooled estimates across MASH F1–F3 patients in the source trials. We applied the same RR multiplier across each fibrosis-stage transition and each MASH resolution transition, assuming proportional effect uniformity across stages, consistent with the assumption underlying the Chhatwal et al. SOC model, which similarly applies stage-independent resolution probabilities.

eTable 1: Base-case results for 10-year time horizon.

| Treatment | Cost ($) | QALYs | Incremental cost ($) | Incremental QALYs | ICER ($/QALYs) |
| --- | --- | --- | --- | --- | --- |
| SoC | 31,375 | 5.73 | - | - | - |
| Resmetirom | 465,529 | 6.77 | 434,154 | 1.04 | 418,006 |
| Semaglutide | 142,684 | 6.54 | 111,309 | 0.81 | 137,422 |
| Tirzepatide | 115,084 | 7.27 | 83,709 | 1.54 | 54,405 |

eTable 2: Base-case results for the normalization scenario over the life-time horizon.

| Treatment | Cost ($) | QALYs | Incremental cost ($) | Incremental QALYs | ICER ($/QALYs) |
| --- | --- | --- | --- | --- | --- |
| SoC | 54,947 | 11.98 | - | - | - |
| Resmetirom | 953,386 | 14.22 | 898,438 | 2.24 | 401,080 |
| Semaglutide | 294,819 | 13.66 | 239,871 | 1.67 | 142,961 |
| **Tirzepatide** | **244,632** | **15.32** | **189,684** | **3.34** | **56,755** |

eTable 3: Maximum annual drug price at which each pharmacotherapy would be cost-effective versus standard of care at three willingness-to-pay thresholds

|  | Maximum annual drug price ($) | | |
| --- | --- | --- | --- |
| Drug | WTP 50K/QALY | WTP_100K | WTP_150K |
| Resmetirom | 12,560 | 23,007 | 33,455 |
| Semaglutide | 10,813 | Meets threshold at current WAC | Meets threshold at current WAC |
| Tirzepatide | 14,844 | Meets threshold at current WAC | Meets threshold at current WAC |

eTable 4: Base-case results for a conservative early MASLD and Non-MASH (F1-F2) states.

| Treatment | Cost ($) | QALYs | Incremental cost ($) | Incremental QALYs | ICER ($/QALYs) |
| --- | --- | --- | --- | --- | --- |
| SoC | 54,947 | 11.33 | - | - | - |
| Resmetirom | 1,003,691 | 14.08 | 948,743 | 2.74 | 346,030 |
| Semaglutide | 298,042 | 13.77 | 243,094 | 2.44 | 99,595 |
| Tirzepatide | 245,218 | 14.76 | 190,270 | 3.43 | 55,458 |

eTable 5: Two-way sensitivity analysis of MASH resolution and fibrosis improvement risk ratios.

| Strategy vs SoC | Scenario | Risk ratio (MASH resolution) | Risk ratio (fibrosis improvement) | Incremental cost | Incremental QALY | ICER |
| --- | --- | --- | --- | --- | --- | --- |
| **Resmetirom** | **base** | **2.54** | **1.64** | **948,743** | **3.47** | **273,445** |
| Resmetirom | low_low | 1.87 | 1.27 | 925,574 | 2.71 | 341,222 |
| Resmetirom | low_high | 1.87 | 2.20 | 959,572 | 3.39 | 282,942 |
| Resmetirom | high_low | 3.55 | 1.27 | 942,132 | 3.65 | 258,280 |
| Resmetirom | high_high | 3.55 | 2.20 | 972,254 | 4.21 | 231,078 |
| **Semaglutide** | **base** | **1.87** | **1.51** | **243,095** | **3.04** | **80,076** |
| Semaglutide | low_low | 1.60 | 1.23 | 240,231 | 2.52 | 95,351 |
| Semaglutide | low_high | 1.60 | 1.90 | 245,889 | 3.11 | 79,097 |
| Semaglutide | high_low | 2.24 | 1.23 | 240,663 | 3.06 | 78,753 |
| Semaglutide | high_high | 2.24 | 1.90 | 246,299 | 3.59 | 68,667 |
| **Tirzepatide** | **base** | **4.65** | **1.77** | **190,271** | **4.46** | **42,705** |
| Tirzepatide | low_low | 2.31 | 1.17 | 186,752 | 3.04 | 61,417 |
| Tirzepatide | low_high | 2.31 | 2.94 | 194,940 | 4.11 | 47,481 |
| Tirzepatide | high_low | 10.66 | 1.17 | 185,592 | 4.63 | 40,106 |
| Tirzepatide | high_high | 10.66 | 2.94 | 193,976 | 5.27 | 36,812 |

eFigure 4: One-way sensitivity of incremental cost-effectiveness ratio to annual treatment discontinuation rate for resmetirom, semaglutide, and tirzepatide versus standard of care.

eFigure 5: ICER sensitivity to treatment-duration cap.

**References**

1.	Chhatwal J, Dalgic OO, Chen W, et al. Analysis of a Simulation Model to Estimate Long-term Outcomes in Patients with Nonalcoholic Fatty Liver Disease. *JAMA Network Open*. 2022;5(9)doi:10.1001/jamanetworkopen.2022.30426

2.	Souza M, Al-Sharif L, Antunes VLJ, Huang DQ, Loomba R. Comparison of pharmacological therapies in metabolic dysfunction–associated steatohepatitis for fibrosis regression and MASH resolution: Systematic review and network meta-analysis. *Hepatology*. 2025:10.1097/HEP.0000000000001254. doi:10.1097/hep.0000000000001254

3.	Dias S, Welton NJ, Sutton AJ, Ades A. Evidence synthesis for decision making 5: the baseline natural history model. *Medical Decision Making*. 2013;33(5):657-670.

4.	Gidwani R, Russell LB. Estimating transition probabilities from published evidence: a tutorial for decision modelers. *Pharmacoeconomics*. 2020;38(11):1153.
